# Critical design choices in healthcare simulation education: a 4C/ID perspective on design that leads to transfer

**DOI:** 10.1186/s41077-023-00242-7

**Published:** 2023-02-24

**Authors:** Jimmy Frerejean, Jeroen J. G. van Merriënboer, Claire Condron, Ulrich Strauch, Walter Eppich

**Affiliations:** 1 School of Health Professions Education (SHE), Faculty of Health, Medicine and Life Sciences, Maastricht University, Maastricht, the Netherlands; 2 Simulation Center Maastricht University Medical Center+, Maastricht, the Netherlands; 3 RCSI SIM Centre for Simulation Education and Research, RCSI University of Medicine and Health Sciences, Dublin, Ireland; 4 Department of Intensive Care, Maastricht University Medical Center +, Maastricht, the Netherlands

**Keywords:** Simulation-based education, Simulation-enhanced education, Instructional design, Transfer of learning, 4C/ID

## Abstract

**Background:**

Healthcare simulation education often aims to promote transfer of learning: the application of knowledge, skills, and attitudes acquired during simulations to new situations in the workplace. Although achieving transfer is challenging, existing theories and models can provide guidance.

**Recommendations:**

This paper provides five general recommendations to design simulations that foster transfer: (1) emphasize whole-task practice, (2) consider a cognitive task analysis, (3) embed simulations within more comprehensive programs, (4) strategically combine and align simulation formats, and (5) optimize cognitive load. We illustrate the application of these five recommendations with a blueprint for an educational program focusing on simulation activities.

**Conclusions:**

More evidence-informed approaches to healthcare simulation might require a paradigm shift. We must accept that a limited number of simulations is not enough to develop complex skills. It requires comprehensive programs that combine simulation sessions with workplace learning.

## Background

Simulation-based education (SBE) in the health professions uses immersive techniques to interactively replicate substantial aspects of the real world [20]. In most instances, SBE provides learners with opportunities to acquire a set of competencies, ultimately leading to the transfer of learning, that is, the application of said competencies in new situations in the workplace. However, even experienced instructional designers find this aspect challenging. Successful transfer requires attention to several key steps: (a) a thorough analysis of content, learners, and the context, (b) the design of a coherent blueprint based on solid instructional theories; (c) the development of high-quality teaching and learning activities and materials; (d) the implementation at many levels in the organization; and (e) a critical evaluation of all of the above (i.e., the ADDIE approach, see [47]).

Instructional theories provide helpful guidelines to design effective instruction. Examples include the Four-Component Instructional Design model (4C/ID; [57]) and the First Principles of Instruction [37]. In this paper, we present five recommendations for designing programs to promote learning transfer. We illustrate these recommendations using a worked example of a 4C/ID-based blueprint using the evaluation and management of patients with respiratory distress as a complex skill. Finally, we discuss some general implications.

### Transfer of learning

According to 4C/ID, two main processes promote transfer of learning. Strongly simplified, we can label them as *variation* and *repetition*. Variation refers to variability of practice [32, 56]. In clinical practice, each set of circumstances is unique and specific to the context. Patients, diagnoses, and interventions are different. By replicating this variability in educational programs, we train learners to apply their knowledge flexibly and adapt their approach to specific tasks at hand. *Nonrecurrent aspects* of complex skills are performed differently in different professional tasks, such as diagnosing, clinical reasoning, dealing with complications, or communication. These skills often involve flexible problem-solving, decision-making, or reasoning appropriate to the situation at hand. To perform them successfully, learners require a broad and highly organized knowledge base that enables them to generate potential solutions. To develop this knowledge base, or *construct cognitive schemas*, learners must engage with varying learning tasks that stimulate them to abstract away from these concrete experiences or examples (i.e., *inductive learning*). Schema construction is also promoted by studying supportive materials and linking new information to existing knowledge (i.e., *elaboration*). Learners then transfer their learning by applying the rich and integrated schemas developed during training to improve their performance in novel tasks and situations. The richer and more integrated the schemas, the more likely that transfer occurs.

Whereas variation is essential for nonrecurrent aspects, repetition is essential for recurrent aspects. *Recurrent aspects* are performed identically in different variations of professional tasks (e.g., performing a physical examination or using a surgical instrument). Because they are identical, they can be trained repeatedly until their performance becomes quick, efficient, and sometimes even automated. The primary learning process here is *schema automation*, stimulated through specific how-to instruction combined with repetitive practice of recurrent aspects. Repeated practice links specific situations to specific actions by creating cognitive IF–THEN rules in memory (i.e., *rule formation*). The more often these rules are practiced, the quicker and easier they will be activated in the future (i.e., *strengthening*). Here, transfer of learning manifests when cognitive rules developed during training improve the accuracy and speed of the same aspects in a new task. The more often the recurrent aspect is practiced, the stronger and more refined the rules become. Thus, transfer becomes more likely.

**Fig. 1:**
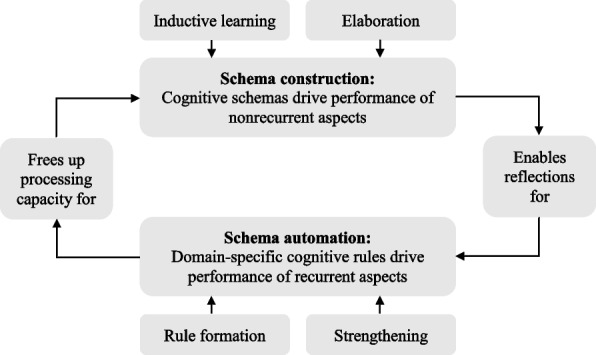
The relationship between the two transfer mechanisms

These two interrelated transfer mechanisms work in parallel, as shown in Fig. 1 [55]. Developing rich cognitive schemas leads to a broad knowledge base that enables learners to reflect on their performance and learn from their successes and failures, extending and refining their cognitive schemas in the process. Practice and reflection also lead to the automation of schemas that make the performance of recurrent aspects fast and effortless, reducing mistakes and freeing up cognitive resources. Learners can then better apply these resources to nonrecurrent skills requiring reasoning and problem-solving, completing the cycle.

In summary, achieving transfer relies on two parallel mechanisms: (a) driving schema construction to allow learners to perform unfamiliar task aspects in new situations, which requires *variation*, and (b) fostering schema automation to enable learners to effortlessly perform familiar aspects of new tasks, which requires *repetition* [26].

We now provide five recommendations for designing healthcare simulation education that fosters transfer of learning. The recommendations are a mix of theory-informed prescriptions stemming from 4C/ID and practical guidelines for applying them. Note that there are no quick strategies that clinicians can apply in their design and teaching tomorrow. Instead, these recommendations offer a first step toward better frameworks and a deeper fundamental understanding of making instructional design decisions.

#### Recommendation 1: Emphasize whole-task practice

Consider the complex skill of assessing and managing pediatric patients with respiratory distress who present to the emergency department. These situations require clinicians to coordinate several component or “constituent” skills, such as rapidly assessing the patient’s appearance, performing a focused history and physical examination, considering triggering factors, initiating appropriate therapy, communicating with caregivers, and collaborating with other medical staff—all while dealing with a potentially stressful environment. In a whole-task approach, learning is centered on realistic tasks that require learners to practice these different aspects in realistic relation to each other. This approach promotes *complex learning*, in which learners integrate knowledge, skills, and attitudes and coordinate required constituent skills as they would during real-life clinical tasks. In other words, they practice the nonrecurrent aspects (e.g., clinical reasoning) and the recurrent aspects (e.g., clinical examinations) in the same task, and sometimes even simultaneously (i.e., communicating with caregivers *while* performing a physical exam).

By focusing on authentic professional tasks in simulation activities, educators avoid *compartmentalization*, which occurs when learners work on knowledge, skills, and attitudes separately. Unfortunately, compartmentalization is widespread: knowledge is addressed in lectures, skills are trained in the skills lab, and attitudes are developed with role-plays. As a result, learners lack opportunities to integrate all three domains as they must in clinical practice, simultaneously applying their knowledge while performing skills and demonstrating attitudes. Whole-task practice also avoids *fragmentation*, or the disconnected training of isolated constituent skills (i.e., part-task training). This pitfall is obvious when “technical” and “non-technical” skills are trained separately, even though they are performed concurrently in real-life tasks (e.g., [5, 9]). Training parts of a task while neglecting others creates problems because whole tasks represent more than the sum of their parts. Research demonstrates that whole-task training is more effective than part-task training for fostering transfer of learning to new situations [30, 62].

The 4C/ID model recommends using increasingly complex whole tasks as the backbone of learning programs to develop these integrated competencies. Part-task training then *complements* whole-task training instead of the other way around, a typical sequence in traditional curricula. This way, learners first confront whole tasks, thus learning why they must train specific constituent skills in isolation. Educational programs promote transfer of learning when they address the necessary coordination and integration by alternating between whole-task practice and part-task practice as complexity increases [16, 18, 19]. The workplace represents an ideal place to “zoom out” to the whole task. For example, surgical trainees receive supervised whole-task practice in the operating room, then “zoom in” and practice part-tasks on a box trainer, and then “zoom out” to integrate these constituent skills in the operating room. Thus, part-task training complements whole-task training. Designers should consider this integration of part-task and whole-task training and avoid presenting part-task practice as isolated events.

#### Recommendation 2: Consider a cognitive task analysis

Educators frequently conduct needs assessments when developing simulation programs to reveal gaps between actual and desired performance. They then formulate lists of learning outcomes to fill those gaps and select instructional methods to reach each outcome [44]. For example, cognitive learning outcomes (e.g., clinical reasoning) are addressed with virtual patients [12], affective learning outcomes (e.g., empathic communication) are taught with human simulation [39], and psychomotor skills (e.g., operating ultrasound devices) are taught with part-task simulations [38].

**Fig. 2:**
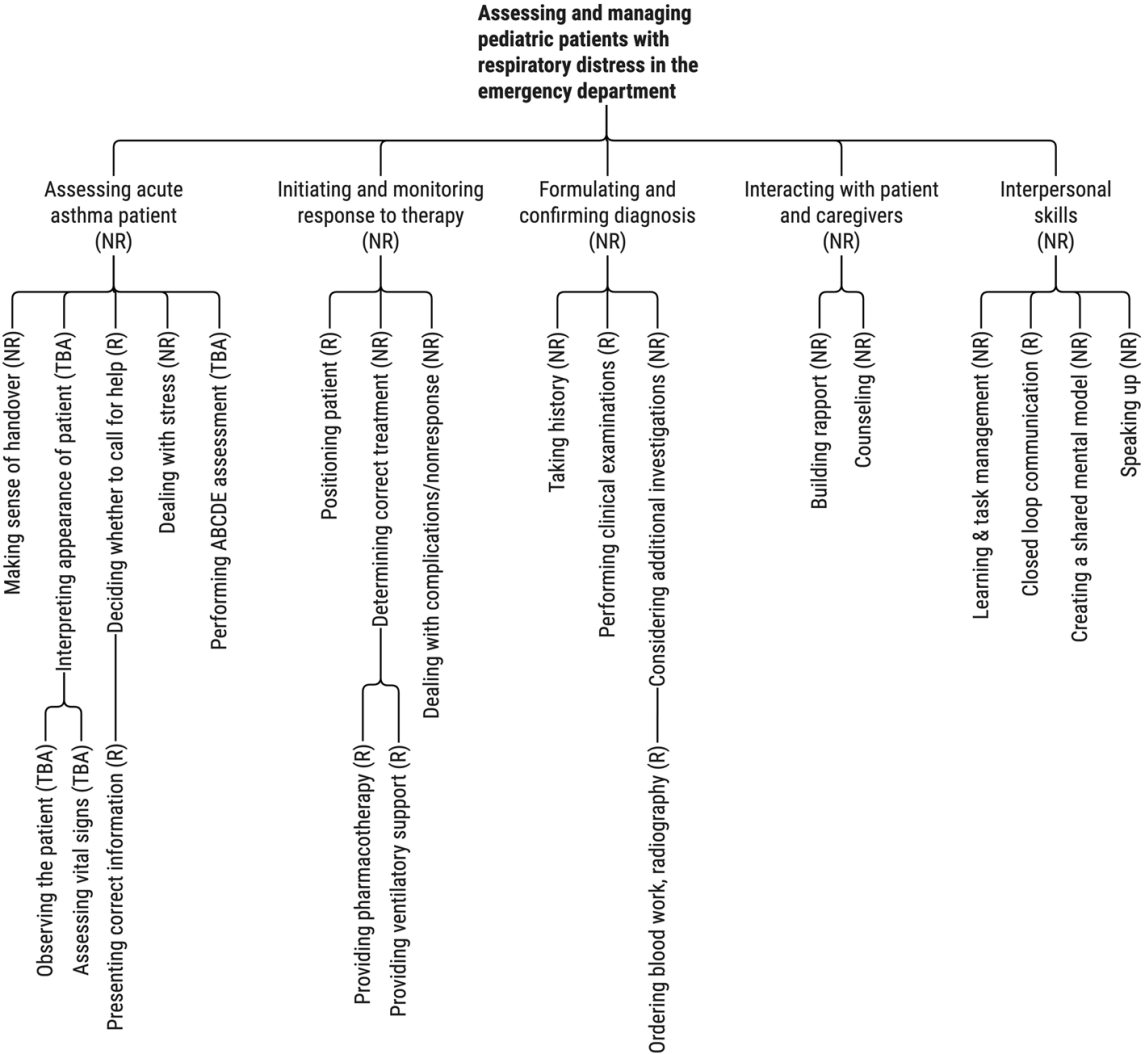
A condensed skill hierarchy for “assessing and managing pediatric patients with respiratory distress in the emergency department.” NR, nonrecurrent; R, recurrent; TBA, to-be-automated

While needs assessments reveal essential learning outcomes, they may encourage part-task approaches that focus only on targeted outcomes while ignoring other aspects of the whole task (i.e., risking compartmentalization and fragmentation). Thus, a needs assessment may not provide all the necessary information about which nonrecurrent and recurrent skills learners must coordinate in a whole task. In addition, evidence shows that clinical experts who teach recall only about 30% of their automated decisions and strategies, leading them to fill these memory gaps with perhaps faulty assumptions when explaining procedures to learners [10]. A more reliable approach is *cognitive task analysis* (CTA). Many types of CTA exist, but the general approach involves document study, observations, and detailed interviews with professional task performers, subject-matter experts, and expert teachers to deconstruct professional tasks into their constituent skills. This deconstruction results in a skill hierarchy that visualizes the constituent skills necessary to perform the complex skill (see Fig. 2). Further knowledge elicitation techniques uncover the required cognitive strategies (i.e., systematic approaches to carry out the task) and the domain knowledge needed for the performance of nonrecurrent skills, and the cognitive IF–THEN rules needed for the performance of recurrent skills. A thorough description of CTA is outside the scope of this paper, but Clark et al. [11], Van Merriënboer and Kirschner [59], and Tjiam et al. [53] offer good starting points.

A CTA and accompanying skill hierarchy enable the design of learning tasks that require the coordination of logical combinations of constituent skills, just like real-life tasks. Designers can also describe and classify the desired exit behavior for each constituent skill. *Nonrecurrent* skills require cognitive schemas for reasoning and decision-making because they are different each time, demanding variation. *Recurrent* skills that are similar across tasks and for which cognitive rules can be formed by the end of the training are labeled recurrent, requiring repetition. Critical recurrent skills that must be automated by the end of the training are labeled *to-be-automated recurrent*. This classification process allows designers to design whole-task practice with the required variation and repetition for the respective aspects.

#### Recommendation 3: Embed SBE in more comprehensive programs

We do not recommend basing an educational program entirely and solely on simulations (i.e., SBE). Instead, a thorough needs analysis and CTA provide primary inputs for the content, structure, and media use, meaning that a well-designed program often includes whole-task practice in both simulated and workplace settings. Educators often overestimate the contribution of single simulation sessions in terms of deep learning or transfer of learning, even when they include high-quality debriefing afterward. A short series of simulations provide insufficient practice and variation in task characteristics necessary to prepare learners for the variability of clinical work. Competency development takes time! Accordingly, designers should not view isolated simulations as a complete training program but as a group of ingredients that should be mixed with other ingredients to create a sophisticated dish.

According to 4C/ID, a complete training program includes four components. The program’s backbone consists of whole learning tasks (component 1). Many learning tasks can be simulations, from paper-based case studies to role-plays, simulated patient encounters, or immersive training with simulation mannequins, but also real-life professional tasks. Supportive information (component 2) helps learners with nonrecurrent aspects. This information can be presented in lectures, workshops, demonstrations, observations, readings, podcasts, e-learning modules, and AR or VR content. Learners study these materials before or during whole-task training. Procedural information (component 3) is presented just-in-time during the learning tasks to help with recurrent aspects. Instructors can provide corrective feedback or materials with how-to instructions, such as job aids, reference cards, manuals, or checklists. Finally, for to-be-automated skills, repeated practice on part-task simulators (component 4), such as box-trainers, can be employed to reach accurate and fast performance.

These four components simultaneously stimulate schema construction and automation, thereby increasing the chances for transfer. In such programs, simulations do not stand alone but are combined with other learning activities to optimally support competency development. Following this line of reasoning, *simulation-enhanced education* would be a more appropriate term than *simulation-based education*. Moreover, transfer is much more likely when combining simulations with workplace tasks, such as observations, debriefing participation, or guided practice with support from peers or supervisors [21, 43]. This combination of workplace learning and simulation promotes transfer learning from simulations to the workplace and helps learners bring relevant work experiences to simulations.

#### Recommendation 4: Combine and align different simulation activities

Specific simulation activities might be effective for whole-task practice, others for working on nonrecurrent aspects, and yet others for recurrent aspects and aspects that must be automated. Therefore, designers should combine simulation activities so learners may optimally develop, practice, and refine their competencies. These complicated design decisions involve balancing key considerations:

- Effectiveness: What is the best way to attain the competencies necessary for whole task performance?
- Efficiency: What does this activity cost in terms of resources such as development time, budget, and personnel?
- Appeal: Will my learners and teachers enjoy this activity?

Thus, educators should not ask, “how can we use the high-fidelity mannequin?” or “what to do with virtual reality?”. The more relevant question is, “which simulation formats are most appropriate to achieve our educational goals in our current situation (e.g., time, budget, staff availability)?”. The decision about particular simulation formats should deliberately balance effectiveness, efficiency, and appeal in the particular context (see also [27]).

Suppose designers seek a method to teach surgical skills and consider adding a virtual reality simulation to the program. In terms of effectiveness, the main question is: Does this VR simulator add opportunities for whole-task practice? Can learners combine nonrecurrent aspects (e.g., clinical reasoning, communications) and recurrent aspects (e.g., making incisions)? Does it increase variability of practice? Can we align the fidelity and challenge for different learners? Can it be used for automating recurrent skills? What is its added value compared to our program’s other instructional methods? While most VR simulators can effectively immerse the learner in an operating room, they sometimes lack haptics and tactile sensations essential for surgical interventions. Effectiveness might be high for some aspects or target groups but low for others.

Regarding efficiency, the main question is: Are there sufficient resources to add this to our program? Available budget, staffing, facilities, student group sizes, and organizational and technical support factor into the cost–benefit analysis. Finally, in terms of appeal, the main question is: Will learners—and staff—enjoy learning with this simulation? Appealing instructional methods may be used more often, provide motivation, and limit fatigue from redundancy in teaching methods.

By systematically considering effectiveness for whole and part-task training, efficiency, and appeal, designers can compare the strengths and weaknesses of different combinations of simulation formats in their programs. Increasing one aspect (e.g., effectiveness) in this “iron triangle” often means sacrificing one of the other two [22]. This triangle illustrates how small changes in context, such as different levels of prior knowledge or a change in the number of students, lead to entirely different design decisions.

#### Recommendation 5: Optimize cognitive load

Although effective learning arises from pushing learners to the edge of their comfort zones, extreme simulation scenarios stack several unlikely complications and disasters to push learners well beyond their limits. This can easily overload learners’ working memory, hampering their learning [48, 61]. The 4C/ID model is firmly based on cognitive load theory (CLT), which recommends attending to cognitive overload and ensuring that activities contribute to learning instead of detracting from it. The cognitive requirements of learning activities should not exceed working memory capacity. Cognitive load theory includes many guidelines for instructional design [17, 61] and has inspired a Cognitive Theory of Multimedia Learning [34] that guides the design of multimedia materials [23]. CLT has been applied to SBE to optimize cognitive load (see [17, 45, 50]), and guidelines exist to increase learning transfer [36]. We distill three important implications for the design of SBE that underpin 4C/ID design principles:

First, the *intrinsic load* brought on by the task itself should be managed. Whereas overly challenging tasks induce cognitive overload, boring or easy tasks (e.g., repetitive part-task training) can cause underload and subsequent learner disengagement. To avoid cognitive overload, complexity should start low and gradually increase once learners master lower-complexity tasks (i.e., a mastery approach [35]). To avoid underload, challenge can be increased by adding time pressure or introducing more whole-task practice. Second, *extraneous load* unrelated to the task and detrimental to learning should be minimized. Support and guide learners when complexity increases by providing modeling examples, imitation tasks, worksheets, or coaching. Avoid unclear or misleading instructions, ambiguous goals, or distractions that do not occur in real-life tasks.^1^ Third, use freed-up cognitive resources to induce *germane load* by introducing activities that contribute to schema construction and automation, such as having learners reflect on variations of tasks (e.g., in a debriefing) and compare different strategies, or by providing cognitive feedback. These activities present desirable difficulties [6, 15]. Increasing germane load is only possible if current intrinsic and extraneous load levels remain within the bounds of the available working memory. If learners already face high demands, adding desirable difficulties will likely lead to cognitive overload and hamper learning.

We should note the challenges in measuring different types of cognitive load. Researchers use measurements such as rating scales [29, 41], dual-task methodology [42], or physiological measurements such as EEG [3] or pupillometry [54]. Educators cannot objectively monitor cognitive load but can make informed inferences. For example, failure or complaints about complexity could indicate too high intrinsic load. Insecurity, struggle, distraction, or frustration could indicate too high extraneous load. Boredom and disengagement might indicate underload. And low levels of learning and transfer despite performing authentic tasks might indicate a lack of germane load. These findings can then give the designer directions for redesigning the program.

### An example blueprint

Using the 4C/ID model [55, 57], we developed a blueprint for a training program for “managing pediatric patients with respiratory distress in the emergency department” that illustrates an application of our recommendations (see Table 1). The blueprint describes a part of a hypothetical undergraduate curriculum, including preclinical and clinical phases, and illustrates how the recommendations *could* be applied in one context rather than prescribing how they *should* be applied in all contexts.

**Table 1:**
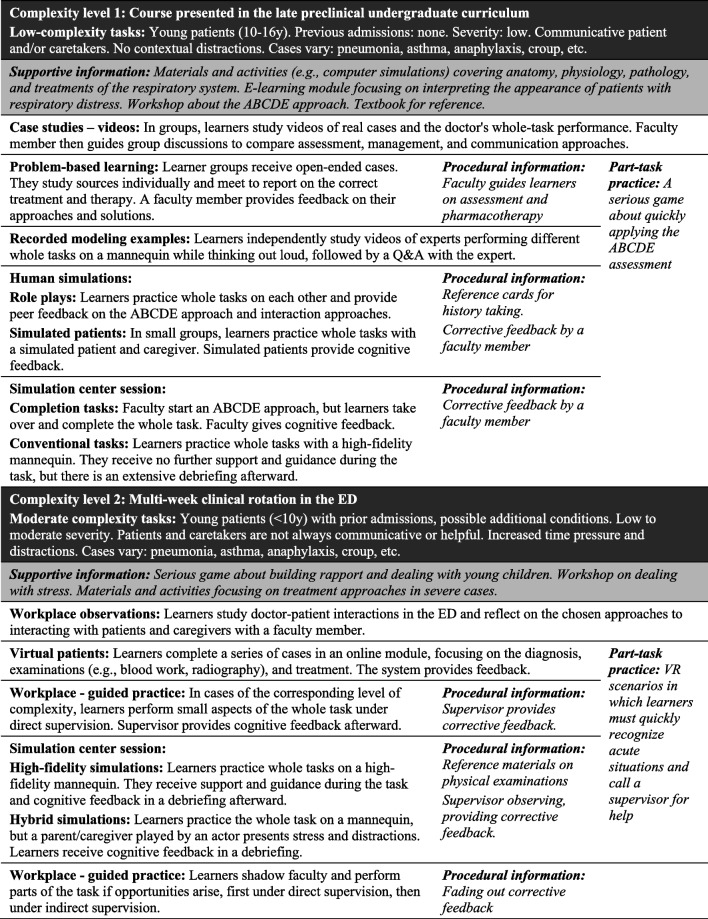
4C/ID training blueprint for “managing pediatric patients with respiratory distress in the emergency department”

In line with our first recommendation, the blueprint contains whole tasks requiring integration of knowledge, skills, and attitudes, and coordination of the recurrent and nonrecurrent skills shown in Fig. 2. Learning tasks (i.e., 4C/ID component 1) are performed in the described order and are grouped into two levels of complexity, increasing from low to moderate, as indicated by the black rows. We also included part-task training (i.e., 4C/ID component 4), zooming in on to-be-automated recurrent skills shown in Fig. 2. Part-task training *follows* whole-task training and is intermixed with whole tasks.

Following recommendation 2, we base this design on the outcomes of cognitive task analysis and the skill hierarchy presented in Fig. 2. First, we observed and interviewed experienced practitioners, decomposed the complex skill, and labeled the skills as nonrecurrent, recurrent, or to-be-automated. Then, *supportive information* (i.e., 4C/ID component 2) was included for the skills labeled as nonrecurrent. This information refers to lectures, workshops, study groups, demonstrations, reading materials, digital materials, and other materials that help learners construct schemas to systematically approach tasks and support reasoning, problem-solving, and decision-making. These materials are described in the shaded rows labeled “supportive information” and remain available throughout the learning program. Next, *procedural information* (i.e., 4C/ID component 3) was added for skills labeled “recurrent” and refers to “how-to” information presented in manuals for operating tools or software, reference cards, or by an instructor observing the learner and correcting mistakes. Educators present this information just-in-time during learning tasks to help learners apply rules and procedures correctly, with immediate feedback as needed. The blueprint describes this content in the cells labeled “procedural information.” A CTA was essential in designing this blueprint to ensure that learning tasks were grounded in clinical practice.

Recommendation 3 focused on embedding simulation activities in broader programs. A comprehensive program includes simulations and non-simulation activities such as workshops, observations, modeling examples, or readings. Following recommendation 4, a rich mix of simulation formats is used, combining the strengths and mitigating limitations of each. This combination balances individual methods’ effectiveness, efficiency, and appeal. For example, virtual patients efficiently train clinical reasoning in a low-stakes setting but complement human simulation to address communication skills and attitudes not covered in the virtual patient activities. Finally, in line with recommendation 5, appropriate levels of complexity, support, and guidance help avoid cognitive overload and desirable difficulties maximize the desired cognitive load.

This example blueprint targets final-year medical students, but training at the postgraduate level could look similar with some adaptations for dealing with complexity and scaffolding. The workplace does not always offer sufficient exposure to varying tasks for all learners, and there is often less control over task sequence and complexity. This potentially opportunistic learning can be compensated with a deliberate intertwining of workplace-based tasks and simulation tasks: simulations provide sufficient practice in a safe and supported environment, and workplace tasks prepare for independent professional practice. In the workplace, scaffolding could follow an approach based on entrustment decisions: first having learners observe workplace tasks and then gradually transitioning to performance under direct supervision, performance with supervision on request, and performance with supervision post hoc [52].

## Conclusions

We provide five recommendations to inform critical design decisions in simulation programs to promote the transfer of learning. First, emphasizing whole-task practice to stimulate skill integration and coordination avoids problems of compartmentalization and fragmentation. Second, a cognitive task analysis, in addition to a needs assessment, provides a more thorough understanding of the whole complex skill and the context in which it is performed, enabling better design decisions. Third, simulation is essential but not sufficient for developing complex skills, and educators should view simulation as a vital element that must be combined with other ingredients in a comprehensive learning program. Fourth, a logical combination of simulation formats should be guided by three considerations: evaluating their *effectiveness* for reaching different learning outcomes, their *efficiency* in terms of required resources, and their *appeal* to staff and learners in the respective context. Fifth, an optimal level of challenge can be achieved by managing the cognitive load to avoid cognitive overload or underload. When applied correctly, these five recommendations help create learning programs that provide the variation and repetition required for transfer of learning.

Implementing these recommendations may pose challenges. While theories are clear about variation and repetition, there is always tension between what is theoretically optimal and practically feasible. For example, there might not be enough time to do a CTA, not enough staff to guide all learning tasks, or not enough practice opportunities in the clinical setting. In addition, these recommendations are less relevant for short learning interventions focusing on single learning goals that do not require complex learning or do not emphasize transfer. Practical circumstances can always create reasons to deviate from these recommendations, but that should not limit innovation. A move toward more evidence-informed approaches might require a paradigm shift; we must accept that complex skills cannot be trained in a limited number of simulation sessions but require comprehensive educational programs that combine simulation sessions with workplace learning in an integrated training blueprint. These five recommendations are a first step in moving away from traditional “see one, do one, teach one” approaches, muddled theories like andragogy [51], and educational myths like learning styles [28]. The full 4C/ID model offers dozens of evidence-informed guidelines for designing learning programs aimed at complex learning. For the interested reader, Van Merriënboer and Kirschner [59] provide a full description, and Table 2 presents an overview of underpinning theories.

**Table 2:** Theoretical underpinnings of the 4C/ID model

Theories description	Relevance for 4C/ID
**Cognitive load theory** [48, 49, 60] Learning entails cognitively demanding processing, but people have limited processing capacity. Failure to learn can often be attributed to exceeding working memory capacity	Cognitive load management should be a significant consideration when designing instruction. The application of CLT prevents cognitive overload (e.g., by simple-to-complex sequencing of learning tasks with scaffolding) but also frees up cognitive resources (e.g., by automating skills with part-task practice) that can be allocated to learning (e.g., by increasing variability of practice)
**Dual process theories** (e.g., [25], [46]) Describe that cognitive processing can arise in two different ways: an implicit, automatic, unconscious process and an explicit, controlled, conscious process	The performance of complex skills is defined by a combination of controlled processes performed in a variable way across situations and automatic processes performed in a highly consistent way across situations
**Reflective expertise** [40, 58], **Adaptive expertise** [7] A kind of expertise that entails performing familiar aspects of a task automatically so processing resources become available for dealing with unfamiliar aspects of the task	Training should facilitate the simultaneous development of domain-specific procedures for familiar, recurrent task aspects and a rich declarative knowledge base for dealing with unfamiliar, nonrecurrent aspects. Whole-task training helps learners coordinate these different task aspects
**Schema theory** (e.g., [2, 4]) Knowledge is organized in schemas: mental structures or frameworks that help us understand the world and allow problem-solving, decision-making, and reasoning	For nonrecurrent aspects, the development of a rich declarative knowledge base, or schema construction, is facilitated by inductive learning with learning tasks and elaboration of supportive information
**ACT-R** ([1]) Describes that human cognition emerges from a cognitive architecture consisting of six modules. A production system containing domain-specific IF–THEN structures interacts with declarative memory and the other modules to drive behavior	For recurrent aspects, the acquired declarative knowledge is compiled into domain-specific procedures or rules. Repetition strengthens these rules. The rule formation and strengthening processes drive the transition from controlled processing to efficient automatic performance
**Cognitive flexibility theory** [24, 31] Describes that learning from case examples through different conceptual perspectives stimulates flexible interconnection of concepts in the mind	Processing information from multiple viewpoints is recommended to ensure that elaboration takes place
**Deliberate practice** [13, 14] Describes expert performance as the result of individualized training by a qualified teacher who communicates the goal of the training and provides immediate feedback so that the learner can make repeated revised attempts	Deliberate practice relates to part-task practice, which allows the learner to repeatedly practice a recurrent task aspect to automate it while receiving immediate feedback

Transfer also involves more than good instructional design. According to studies, two other factors affect the transfer of learning [8, 33]. First, personal characteristics influence the potential for transfer, such as learners’ motivation, involvement, readiness, and capacities, as well as teachers’ competency and teaching approach. Second, environments can vary from favorable to obstructing. This includes work environments and organizational structure (e.g., availability of workplace supervision and support), but also organizational culture and (human-resource) management (e.g., psychological safety of learners). The way these three categories of factors interact to promote or inhibit transfer depends strongly on the context.

Applications of 4C/ID and other strategies to promote transfer present exciting avenues for further research in healthcare simulation. Investigating the interplay between simulation-enhanced education design and personal and organizational factors will enhance understanding of achieving seamless transitions between simulation environments and the workplace. While many open questions remain for researchers, today’s designers should integrate what is currently known about achieving transfer. We hope this paper inspires simulation educators to recognize current gaps in their approach to simulation-enhanced education while providing concrete guidance to make better-informed design choices. Just like clinical reasoning, developing instructional design skills requires time and practice. New research findings and innovations advance our field, requiring substantial investments in professionalization to remain current. Therefore, we must attend to faculty development initiatives, communities of practice, and other learning opportunities to support designers*.* Collaborations between universities, hospitals, clinics, simulation centers, and other research institutes at national and international levels will catalyze professional development in simulation-enhanced education. Evidence-informed approaches adopted in healthcare should translate to health professions education in general and healthcare simulation in specific.

## Data Availability

Not applicable. The manuscript does not contain any data.

## Abbreviations

SBE: Simulation-based education
4C/ID: Four-component instructional design
ADDIE: Analysis-Design-Development-Implementation-Evaluation
CTA: Cognitive task analysis
CLT: Cognitive load theory

## Footnotes

1 Distractions that are part of real-life tasks, such as beepers going off, noisy environments, or upset family members, should be considered intrinsic load, because they are inherent to the task. These might be left out for novice learners but must be included for advanced learners.
